# Publication barriers and facilitators of Cochrane authors in sub‐Saharan Africa: A mixed‐methods study

**DOI:** 10.1002/cesm.12054

**Published:** 2024-04-03

**Authors:** Idriss I. Kallon, Taryn Young, Tonya A. MacDonald, Anel Schoonees, Joy Oliver, Dachi I. Arikpo, Solange Durão, Emmanuel Effa, Ameer S.‐J. Hohlfeld, Tamara Kredo, Charles S. Wiysonge, Lawrence Mbuagbaw

**Affiliations:** ^1^ Centre for Evidence‐Based Health Care, Division of Epidemiology and Biostatistics, Department of Global Health, Faculty of Medicine and Health Sciences Stellenbosch University Cape Town South Africa; ^2^ Department of Health Research Methods, Evidence and Impact McMaster University Hamilton Ontario Canada; ^3^ Cochrane South Africa South African Medical Research Council Cape Town South Africa; ^4^ Health Systems Research Unit South African Medical Research Council Cape Town South Africa; ^5^ Cochrane Nigeria Calabar Institute of Tropical Diseases Research and Prevention, University of Calabar Teaching Hospital Calabar Nigeria; ^6^ Department of Medicine, Division of Clinical Pharmacology, Faculty of Medicine and Health Sciences Stellenbosch University Cape Town South Africa; ^7^ Vaccine‐Preventable Diseases Programme World Health Organization (WHO) Regional Office for Africa Brazzaville Congo; ^8^ Department of Anesthesia McMaster University Hamilton Ontario Canada; ^9^ Department of Pediatrics McMaster University Hamilton Ontario Canada; ^10^ Biostatistics Unit Father Sean O'Sullivan Research Centre, St Joseph's Healthcare Hamilton Hamilton Ontario Canada; ^11^ Centre for Development of Best Practices in Health (CDBPH) Yaoundé Central Hospital Yaoundé Cameroon

**Keywords:** Cochrane Reviews, mixed‐methods, non‐Cochrane reviews, publication practices, sub‐Saharan Africa

## Abstract

**Background:**

Well‐conducted systematic reviews contribute to informing clinical practice and public health guidelines. Between 2008 and 2018 Cochrane authors in sub‐Saharan Africa were publishing progressively fewer Cochrane Reviews, compared to non‐Cochrane reviews. The objective of this study was to determine what motivated trained Cochrane authors in sub‐Saharan Africa to conduct and publish non‐Cochrane reviews over Cochrane Reviews.

**Methods:**

We conducted a mixed‐methods exploratory sequential study. We purposely selected 12 authors, who had published at least one Cochrane‐ and one non‐Cochrane review, for in‐depth, semi‐structured interviews. We manually coded and analysed the qualitative data using Grounded Theory approach and used the results to inform the survey questions. Subsequently we surveyed 60 authors with similar publishing experience. We analysed the quantitative data using descriptive and inferential statistics.

**Results:**

Facilitators to publish with Cochrane were a high‐impact factor, rigorous research, and visibility. From barriers, the main categories were protracted time to complete Cochrane Reviews, complex title registration process, and inconsistencies between Cochrane Review groups regarding editorial practices. From the survey, authors confirmed rigorous research and reviewing process (84%), high impact factor (77%), and good mentorship (73%). The major barriers included Cochrane's long reviewing process (70%) and Cochrane's complicated title registration (50%). Authors with publishing experience in the previous 10 years at <95 percentile of systematic review publications, there was no significant difference between the medians for publishing with Cochrane (1) and non‐Cochrane (0) reviews, *p* = 0.06. Similarly, for those with publishing experience of ≥95 percentile of systematic review publication there was no significant difference between the medians for publishing with Cochrane (4) and non‐Cochrane (6), *p* = 0.344.

**Conclusion:**

Authors considered the visibility and relevance of Cochrane research as a trade‐off point. They continued publishing with Cochrane despite the barriers that they encountered. However, the concerns raised by many authors are worth addressing.

## 1 BACKGROUND

Systematic reviews (SRs) are important because when they are well‐conducted they provide reliable and comprehensive summaries of evidence on a given question and play an important role in informing clinical practice and public health guidelines [1]. Cochrane, as a global leader in the production of SRs, advocates for researchers and networks to conduct and use SRs in healthcare decision‐making [2, 3]. There have however been some challenges in achieving this goal in low‐ and middle‐income countries (LMICs) [4, 5]. Early in 2019 Cochrane authors who are also part of this study, conducted a bibliometric study of the publication practices of Cochrane authors in sub‐Saharan Africa (SSA). Through Archie, Cochrane's central database for managing contact information and other SR logistics, they identified 655 SSA Cochrane authors from 19 countries. For each SSA author in the leading (first) or senior (last) position of authorship, they searched for all SRs that they had published in the 10 years before April 2019. Of a total of 757 eligible SRs (from 478 authors), 47% were Cochrane Reviews. It was found that between 2008 and 2018 the number of non‐Cochrane reviews steadily increased from 14 to 61 in a year, while Cochrane Reviews increased from 16 to 31 in a year within the same period [6, 7]. These preliminary empirical data suggest that Cochrane authors in SSA are publishing progressively fewer Cochrane Reviews, and this warrants investigation. The bibliometric research also suggests that exploring the barriers and facilitators that authors encounter may help in the design and implementation of initiatives to enhance research capacity building and the growth of Cochrane in SSA [7]. The research capacity building is mainly within the context of conducting well‐designed SRs in LMICs. Strengthening evidence‐based health research methods is essential for informing health policy and improving health outcomes in SSA, especially considering its high burden of disease [7, 8]. The objective of this study was to determine what predisposes trained Cochrane authors in SSA to conduct and publish non‐Cochrane reviews over Cochrane Reviews.

## 2 METHODS

### 2.1 Study setting and design

We conducted a mixed‐methods exploratory sequential study that consisted of two phases: an initial qualitative phase, followed by a quantitative phase. This approach drew on the depth and breadth of data from qualitative and quantitative methods, resulting in richer data and a better understanding of the answers to our research questions [9, 10, 11]. The qualitative questions we sought to answer were:

- How do sub‐Saharan Africa Cochrane authors decide to publish a Cochrane or a non‐Cochrane review?
- How do sub‐Saharan Africa Cochrane authors perceive trade‐offs between Cochrane and non‐Cochrane reviews?

The quantitative questions were:

- What proportion of sub‐Saharan Africa Cochrane authors experience barriers to publishing Cochrane Reviews?
- What are the characteristics of sub‐Saharan Africa Cochrane authors who experience barriers to publishing Cochrane Reviews?
- What factors are associated with publishing non‐Cochrane reviews?

The subsequent mixed methods questions were:

- How could the factors associated with the choice of systematic review type be explained?
- What roles do sub‐Saharan Africa authors play in determining where to publish SRs in which they participated as lead or senior authors?

### 2.2 Sampling and data collection

#### 2.2.1 Qualitative phase

In the qualitative phase, we purposefully selected authors from different SSA geographical zones with different levels of experience in conducting and publishing SRs. Authors were invited by email to take part in a Zoom or telephonic interview. Twenty‐five prospective authors were purposely invited, five declined, eight did not respond. We interviewed the remaining 12 authors who had published at least one Cochrane‐ and one non‐Cochrane SR in the previous 10 years from two groups defined by their publishing experience: those who have <95th percentile (less than 3 SRs), publications and those who have ≥95th percentile (3 or more SRs) publications in the previous 10 years. We did not include those who conducted qualitative evidence synthesis or mixed methods research in the sample.

The individuals who consented to being contacted were approached to set up a convenient time for an in‐depth, semi‐structured interview via Zoom or telephonically. The interview guide included questions that asked participants to explain their work, research experiences, and what influenced their decision to publish a Cochrane or non‐Cochrane review. Probing questions included the kinds of SRs they conducted, the review groups they have worked with and their experiences of mentorship. We used the qualitative data to develop the electronic survey through iterative discussions with the other team members. For instance, under the theme of facilitators to publishing Cochrane in the qualitative data, we used authors' exact words, such as “high impact factor,” as part of the survey questions that reflected the one aspect of publication experiences of Cochrane authors in SSA. See the list of qualitative and quantitative questions under Supporting Information (S1: Qualitative interview guide and S2: Quantitative survey questions). Authors were given the opportunity to provide consent for the interview to be recorded. Each interview lasted about 30 to 60 min. The median time of the interviews was 45 min. The interviews were conducted from March 4, 2021 to April 26, 2021.

#### 2.2.2 Quantitative phase

We invited all 187 Cochrane authors in SSA who had published at least one Cochrane Review and one non‐Cochrane review in the past 10 years as lead or senior author, and who had been identified in the bibliometric study, to participate in a survey. We invited those who participated in the qualitative interviews. A total of 172 authors successfully received an email from the research team to participate in the online survey, 60 authors completed the survey, which was a 35% response rate. Fifteen email addresses could not be reached despite checking possible alternative email addresses.

The survey was pilot‐tested with three SSA Cochrane authors (not invited to take part in the survey) before launching. We collected basic socio‐demographic data such as gender, country of residence, profession, publication history (number of SRs published), number of years as a researcher, and publication experiences for Cochrane and non‐Cochrane reviews. Response rates in electronic surveys are often low [12]. We attempted to optimise response rate by securing alternative email addresses of some authors who were unresponsive to earlier emails or some outdated emails, keeping the survey as short as possible, and leaving the survey open for 2 months with weekly reminders. The survey was conducted from September 2, 2021 to October 31, 2021.

### 2.3 Data analyses

#### 2.3.1 Qualitative

All interviews were transcribed from recordings and manually coded into categories by two independent coders (TM and IK), and compared for consistency. These categories were developed iteratively and ultimately merged into themes that reflected the publication experiences of SSA Cochrane authors. The qualitative data analysis was informed by Grounded Theory [13]. Grounded Theory method was appropriate because we analysed the data devoid of a preconceived theoretical framework. The process was predominantly inductive with no pre‐existing themes and categories. The first step included a line by line coding to develop categories leading to “conceptual components,” which signified our reflections and understanding of the data. The second step of analysis was making connections between categories through comparisons of sets of data and thirdly, integrating the core categories that were tested in the quantitative phase.

#### 2.3.2 Quantitative

The baseline data and outcomes were summarized as counts (percentages) for categorical variables, median <95th percentile and those who have ≥95th percentile for continuous or discrete variables as appropriate depending on the distribution. Factors associated with publication practices and experiences were determined using bivariate analyses and Pearson tests for continuous or discrete variables and Chi‐squared tests of independence for categorical variables. We used Wilcoxon rank sum test to compare the medians for the number of Cochrane and non‐Cochrane reviews published in the previous 10 years. We used IBM SPSS Statistics (Version 27) predictive analytics software to analyse the data. We presented the key results graphically and in tables.

#### 2.3.3 Data integration and management

We drew inferences from both qualitative and quantitative phases, and across phases as was stipulated in the mixed‐methods questions above. Conclusions were drawn from these meta‐inferences using the “Triangulation protocol” method [10]. For instance, we explained more subjective experiences that were useful in qualitative findings that the survey did not capture. We determined that some findings from the qualitative data were not confirmed by authors in the survey. See Supporting Information S3: Good Reporting of A Mixed Methods Study (GRAMMA) checklist.

Audio recordings were transcribed and stored. The data, with identifying information were removed and were securely stored in a password‐protected computers and shared through password protected Dropbox accounts for analysis. Only members of the research team accessed the data.

## 3 RESULTS

### 3.1 Qualitative findings

#### 3.1.1 Authors' demographics and publication profile

Twelve authors were interviewed. Most of the authors were from South Africa (*n* = 8), and the remaining authors were from Nigeria (*n* = 1), Uganda (*n* = 1), Kenya (*n* = 1), and Botswana (*n* = 1). There were eight male and four female authors. Six authors had <95th percentile publication within the previous 10 years and four with ≥95th percentile in the same period. Eight of the authors had 10–20 years research/publication experience and four had more than 20 years research/publication experience.

#### 3.1.2 Facilitators to publish with Cochrane

##### High‐impact factor, rigorous research, and visibility

Many authors maintained that not only did the Cochrane Library have a high impact factor, but most of the research conducted through Cochrane was important, and often helps health policy makers. The Cochrane Library was also the first place most authors searched for evidence; they mentioned that they searched the Cochrane Library to see examples of how rigorous research process was reported.

*“One of those factors is obviously the Cochrane Library itself has a fairly high impact factor and so the reviews are impactful.”* (Author 8, South Africa)

*“To inform clinicians and make decisions…the most robust way as possible and for us, we feel that Cochrane does do that in terms of the rigorous nature in how the research is guided as well as presented.”* (Author 5, South Africa)

*“The visibility of the Cochrane Reviews because I think for many people Cochrane is a kind of a trademark which carries a lot of weight and tends to be the first place that people look for evidence.”* (Author 1, Botswana)

##### Good support; training, and mentorship

Some of the authors reflected that the free resources and trainings helped shape their research capacity. They also regarded themselves being fortunate to receive excellent mentorship from working with experienced authors.

*“What we gain from Cochrane, publishing Cochrane, our budget is free, free training, and free mentorship and a lot of resources, and actually developed I mean, we grew from the Cochrane collaboration.”* (Author 2, Nigeria)

*“I've always have been fortunate…to have excellent mentorship on reviews. When as a junior starting author, I had the mentorship of two very experienced authors.”* (Author 3, South Africa)

*“I was part of the group reviews for Africa programmes around mentorship, and I think that mentorship, the skills I got from that basically were critical…the mentorship I got was excellent.”* (Author 7, Uganda)

#### 3.1.3 Barriers to publishing with Cochrane

##### Protracted time to complete Cochrane SRs and high research output demand

Some authors recounted that one of the key indicators of being a productive researcher was being able to meet certain outcomes set by university departments and faculties. The authors juxtaposed the expected time to complete a SR with the high demand by their universities for research outputs. As a result, authors would publish SRs with other journals that did not take too much time to complete the peer‐review process and publication of a SR.

*“Factors that influence me to do a non‐Cochrane review is that they don't take so much time unlike the Cochrane review.” (Author 1, Kenya)*

*“It is the output within academia…if we don't produce that output it looks like I'm not doing anything. So that is why we have shifted to other journals.”* (Author 5, South Africa)

##### Complex title registration process and inconsistencies between different Cochrane Review groups regarding editorial practices

A few authors mentioned complex processes as barriers. This complexity included the drive towards registering titles linked to funded projects and registering topics that researchers may not have time to complete, limiting other researchers who may have the time to complete the research. They also highlighted conflicting feedback regarding methods from different Cochrane Review groups.

*“The review titles that they agree to register are predetermined by the area of focus of their funders…I think there is a lot of complex influence of peer reviewing….”* (Author 2, Nigeria)

*“I also found that between different review groups, there's often different methods of doing things and sometimes the expectations are a bit different.”* (Author 8, South Africa)

##### A lack of recognition in research collaboration and a lack of transparency in the writing process

Although many authors mentioned that there was adequate support provided to authors, including free training and mentorship, other authors felt their ideas were not always respected when involved in some research collaborations with authors from other regions. In addition, some of them also felt that there was restriction of academic freedom. They needed more freedom in the choice of topic and the direction of registered protocol and needed more clarity regarding editorial practices.

*“Cochrane needs to be true to its principles of promoting diversity…and remove the barriers to people doing Cochrane Reviews and publishing Cochrane Reviews, it needs to be transparent.”* (Author 12, South Africa)

*“To review the protocol…is a process that reduces the freedom of expression…a lot of strong dictation to how the protocol should be written or should go.”* (Author 2, Nigeria)

*“Many people that I have spoken to in sub‐Saharan Africa have felt disrespected, ignored, not supported.”* (Participant 12, South Africa)

#### 3.1.4 Future publication practices and the way forward

##### Cochrane preferred based on impact‐factor and mentoring opportunities

Despite the many barriers explained by some authors, most of them preferred to continue publishing with Cochrane. A few of them highlighted the importance of conducting Cochrane Reviews and their mentoring responsibilities within Cochrane. They would publish with a non‐Cochrane journal when they needed a quick turnaround based on project‐specific timelines. Nonetheless, their preferred publication choice would always be Cochrane.

*“Cochrane is extremely important as a brand and a contributor to healthcare…there is a very important synergism…that are needed for the WHO guidelines…in turn have a huge impact on practice worldwide.”* (Author 11, Botswana)

*“I mentor a lot of students, masters, PhD students and a lot of researchers…So I'm still involved in Cochrane workshops, protocol development workshops, and systematic review workshops.”* (Author 2, Nigeria)

##### Suggestions for the way forward

Many authors made suggestions to improve the publication practices with Cochrane based on the barriers they encountered. The key suggestions included allowing a balance between rigor and speed, increase academic freedom among authors, increase Cochrane Review group staff, centralize the editorial process, and provide oversight and support the functioning of Cochrane Review groups.

*“…it takes so long to publish a Cochrane review…it's important to get more hands on decks that can facilitate or fast‐track the processes… I also feel that maybe the Cochrane review teams are overworked.”* (Author 2, Nigeria)

*“I would say the level of freedom, academic freedom, or ingenuity…to be encouraged, and not to be restrictive. I think Cochrane is too restrictive… I mean, if you reject people's ideas you are limiting or you are reducing, I mean, you are limiting their freedom, you are limiting productivity, you know.”* (Author 2, Nigeria)

*“To have 50 Cochrane review groups organised by topic and get separate funding for it and get people to work within those groups. I think that model has passed its shelf‐life and … I think that there might be a case to be made here for centralising its resources and having a central editorial base.”* (Author 12, South Africa)

### 3.2 Quantitative results

#### 3.2.1 Authors' demographics and publication profile

Sixty of the 172 (35%) authors invited responded to the survey, of which 30 (50%) were male. Most authors, 28 (46.7%), were from South Africa followed by Nigeria, 15 (25%), Kenya, 4 (6.7%), Cameroon, 3 (5%), Malawi, 2 (3.3%), Uganda, 1 (1.7%), Gambia, 1(1.7%), Tanzania, 1 (1.7%), the remaining authors, 5 (8.3%), were authors residing outside Africa. There were 25 (41.7%) academics, 18 (30%) researchers, 15 (25%) clinicians, 1(1.6%) clinician and academic and 1(1.6%) retired academic. The median of <95 percentile publication of Cochrane Reviews was 1, and that of ≥95 percentile publication of Cochrane Reviews was 4. The median of <95 percentile publication non‐Cochrane reviews was 0, and that of ≥95 percentile publication of non‐Cochrane reviews was 6.

#### 3.2.2 Barriers and facilitators to publishing Cochrane reviews

Thirty‐eight authors (63.3%) experienced barriers publishing Cochrane SRs, while 46 (76.7%) experienced facilitators to publishing Cochrane Reviews. Thirty‐six (60%) believed that the Cochrane Review process could be improved, one (1.7%) did not believe that the review process could be improved and 23 (38.3%) were not sure.

#### 3.2.3 Levels of agreement with barriers to publishing Cochrane reviews

**Figure 1:**
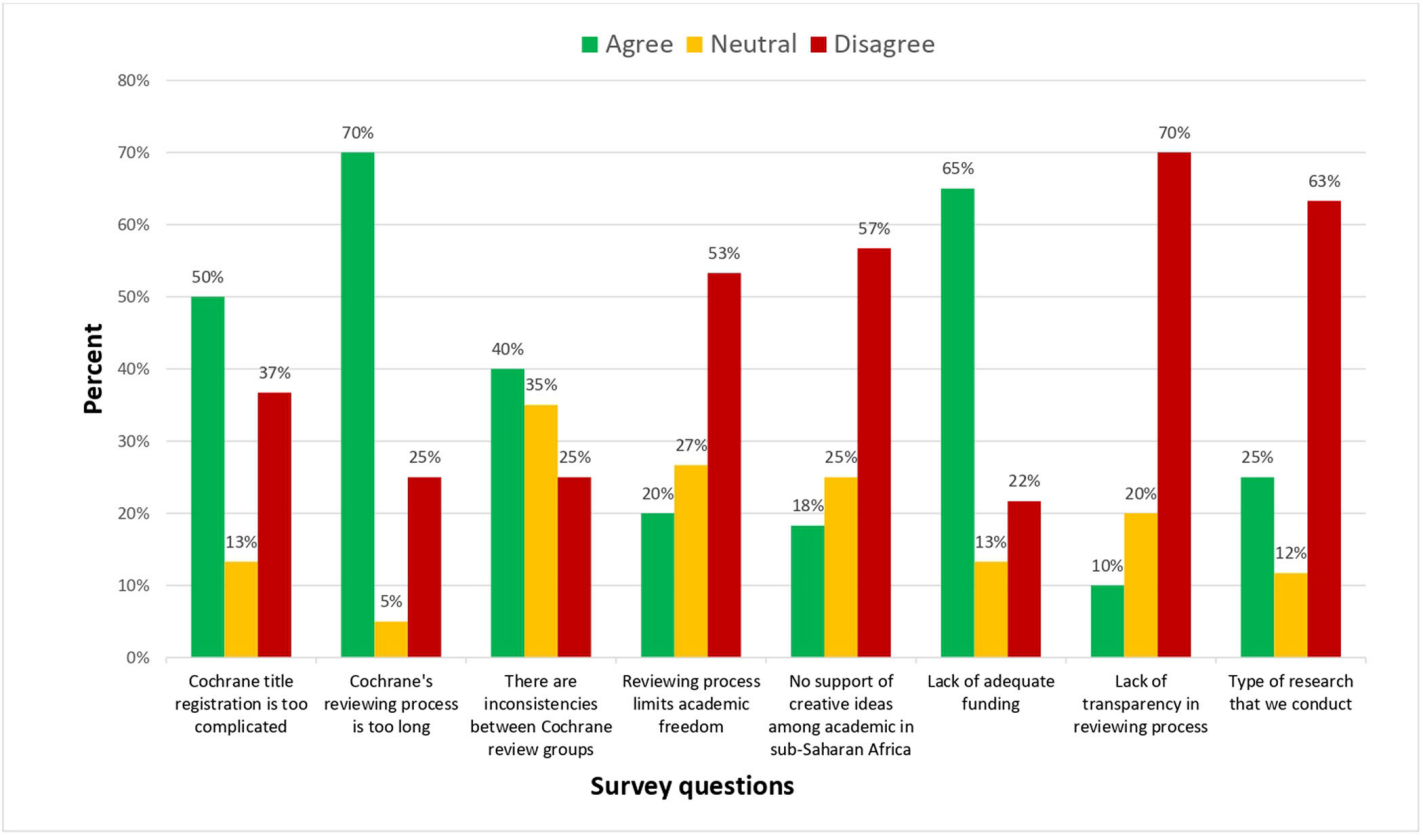
Levels of agreement with barriers to publishing Cochrane Reviews.

Regarding authors' level of agreement regarding specific barriers to publishing Cochrane Reviews, 70% agreed that Cochrane's reviewing process was too long, 50% agreed that Cochrane's title registration was too complicated, and 65% agreed that lack of funding was a barrier. See Figure 1 for the remaining responses, including those authors who remained neutral.

#### 3.2.4 Levels of agreement with facilitators to publishing Cochrane Reviews

**Figure 2:**
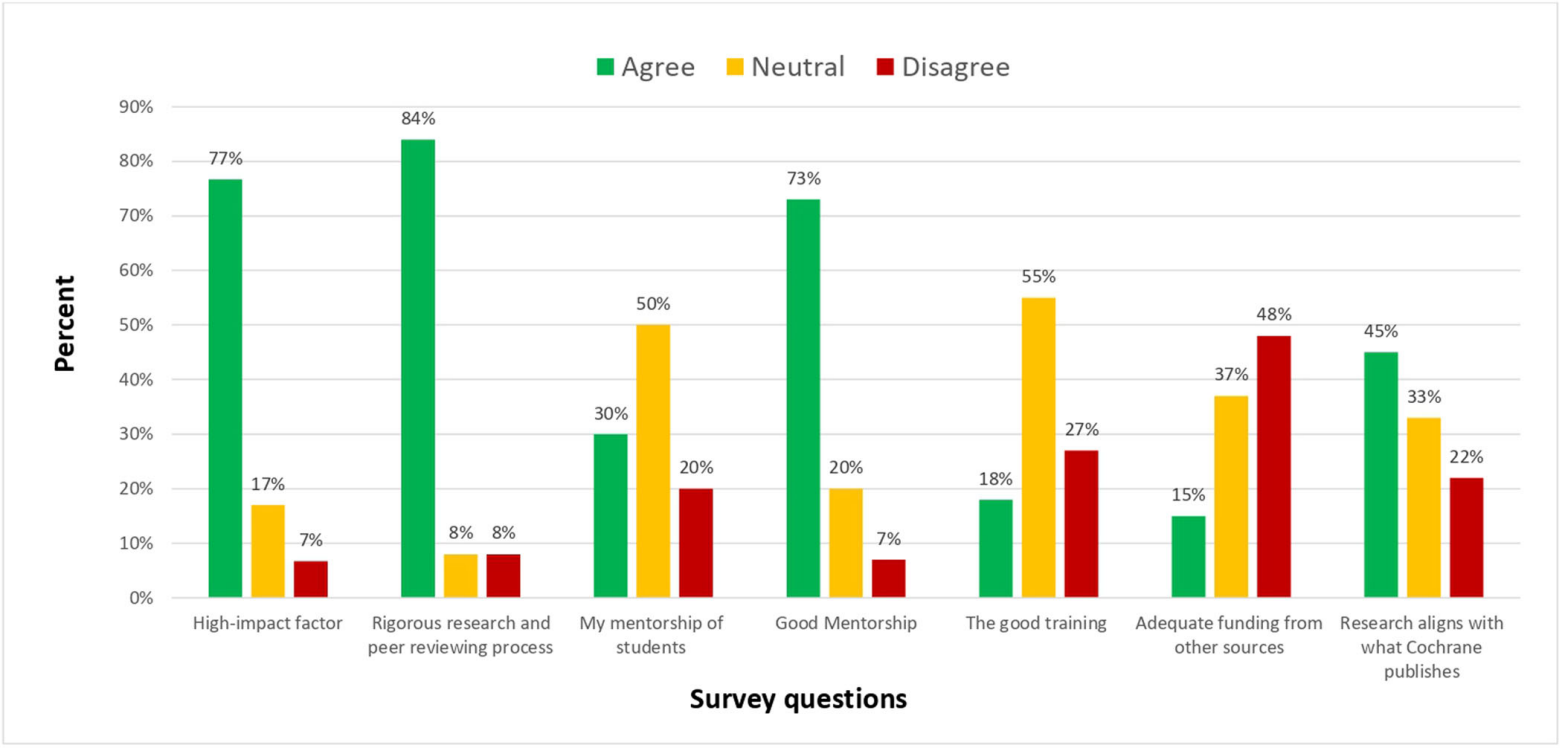
Levels of agreement with facilitators to publishing Cochrane Reviews.

Of all authors who responded, most agreed that the following were facilitators of publishing Cochrane Review: rigorous research and reviewing process (84%), high impact factor (77%), and good mentorship (73%). The remaining levels of agreement with facilitators are found in Figure 2.

#### 3.2.5 Characteristics of authors experiencing barriers and facilitators to publishing Cochrane Reviews

The 30 authors from South Africa and Nigeria made up half (50%) of those who experienced barriers in publishing Cochrane Reviews. Almost the same percentage of authors who experienced barriers have published both Cochrane Reviews, 38 (63.3%) and non‐Cochrane reviews, 38 (64.4%) in the previous 10 years. There was no significant relationship between authors who experienced barriers and publishing Cochrane, 38 (*p* = 0.628) or non‐Cochrane reviews, 38 (*p* = 0.785).

Approximately half of the authors which experienced facilitators were from South Africa and Nigeria, 23 (55%). Interestingly, authors who experienced facilitators published more non‐Cochrane reviews, 46 (78%).

Thirty‐two (53.3%) authors experienced both barriers and facilitators to publishing Cochrane Reviews. There were positive significant relationships between authors who experienced facilitators, but still published more non‐Cochrane reviews (*p* = 0.010) and those who fell within ≥95 percentile (those who have published three or more SRs in the previous 10 years) (*p* = 0.001). There was no significant difference between the medians for publishing Cochrane (*p* = 0.060) and non‐Cochrane (*p* = 0.344) reviews. See Table 1 for the remaining statistics.

**Table 1:** Characteristics of authors experiencing barriers and facilitators to publishing Cochrane Reviews.

	Experience barriers			Experience facilitators		
Variable	Yes	No	*p* Value	Yes	No	*p* Value
Sex: *n* (%)						
Female	19 (31.7)	11 (18.3)	0.999	23 (38.3)	7 (11.7)	0.999
Male	19 (31.7)	11 (18.3)		23 (38.3)	7 (11.7)	
Country of residence: *n* (%)						
South Africa	20 (33.3)	8 (13.3)	0.237	21 (35.0)	7 (11.7)	0.774
Nigeria	10 (16.7)	5 (8.3)		12 (20.0)	3 (5.0)	
Kenya	2 (3.3)	2 (3.3)		3 (5.0)	1 (1.7)	
Cameroon	1 (1.7)	2 (3.3)		2 (3.3)	1 (1.7)	
Malawi	0 (0.0)	2 (3.3)		2 (3.3)	0 (0.0)	
Uganda	1 (1.7)	0 (0.0)		1 (1.7)	0 (0.0)	
Gambia	0 (0.0)	1 (1.7)		0 (0.0)	1 (7.1)	
Tanzania	0 (0.0)	1 (1.7)		1 (1.7)	0 (0.0)	
Outside Africa	4 (6.7)	1 (1.7)		4 (6.7)	1 (1.7)	
Profession: *n* (%)						
Academic	14 (23.3)	11 (18.3)	0.490	17 (28.3)	8 (57.1)	0.532
Researcher	11 (18.3)	7 (11.7)		15 (25.0)	3 (21.4)	
Clinician	11 (18.3)	4 (6.7)		12 (20.0)	3 (21.4)	
Other	2 (3.3%)	0 (0.0)		2 (3.3)	0 (0.0)	
Number of Cochrane Reviews in the past 10 years	38 (63.3)	22 (36.7)	0.628	46 (76.7)	14 (23.3)	0.189
Number of Cochrane Reviews in the past 10 years (<95 percentile publication)	28 (62.2)	17 (37.8)	0.215	33 (73.3)	12 (26.7)	0.653
Number of Cochrane Reviews in the past 10 years (≥95 percentile publication)	10 (67.7)	5 (33.3)	0.822	13 (86.7)	2 (13.3)	0.450
Number of non‐Cochrane reviews in the past 10 years	38 (64.4)	21 (35.6)	0.785	46 (78.0)	13 (22.0)	0.010
Number of non‐Cochrane reviews in the past 10 years (<95 percentile publication)	24 (63.2)	14 (36.8)	0.290	30 (78.9)	8 (21.1)	0.219
Number of non‐Cochrane reviews in the past 10 years (≥95 percentile publication)	14 (66.7)	7 (33.3)	0.964	16 (75.0)	5 (25.0)	0.001

#### 3.2.6 Suggestions for the way forward

Drawing on the key suggestions made by authors in the qualitative interviews, there were agreements with the survey. Seventy‐one percent recommended that there should be some form of oversight to enhance the consistency in processes between Cochrane Review groups. Sixty‐two percent suggested that authors must have academic freedom in terms of the choice of topic, design, and structure of SRs; 52% suggested to increase the number involved in reviewing submissions; 42% suggested to centralise peer‐reviewing processes and 30% suggested to provide more funding to review groups.

## 4 TRIANGULATING KEY QUALITATIVE AND QUANTITATIVE FINDINGS

Under facilitators to publishing Cochrane Reviews, the qualitative theme was *high‐impact factor and rigorous research*, which 77% of authors in the survey agreed that high‐impact factor was a facilitator to publishing a Cochrane SR while 84% agreed to rigorous research process being a facilitator. Under barriers to publishing Cochrane's SR category, the qualitative theme was *protracted time to complete Cochrane SR*, which 70% of authors in the survey agreed. See Table 2 for the remaining results.

**Table 2:** Triangulating key qualitative and quantitative findings.

Qualitative themes (facilitators)	Quantitative results (facilitators)
*High‐impact factor and rigorous research*	Seventy‐seven percent of authors in the survey agreed that high‐impact factor was a facilitator to publishing a Cochrane SR while 84% agreed to rigorous research process
Good support: mentorship and good training	Seventy‐three percent agreed to mentorship and only 18% agreed to good training
**Qualitative themes (barriers)**	**Quantitative results (barriers)**
Protracted time to complete Cochrane SRs	Seventy percent of authors agreed to protracted time to complete Cochrane SRs
Complex title registration process and inconsistencies between different review groups regarding editorial practices	Fifty percent of authors agreed that the title process was too complicated, and 40% agreed to consistencies between editorial groups
A lack of recognition in research collaboration and a lack of transparency in the writing process	Fifty‐seven percent of authors disagreed that there was lack of recognition or creative ideas among SSA authors.
	70% disagreed that there was a lack of transparency in the writing process

## 5 DISCUSSION

Drawing on research showing that Cochrane authors in SSA are publishing more SRs in non‐Cochrane journals [4, 5, 6] we sought to understand why authors make this decision. Authors who experienced facilitators to publishing Cochrane Reviews and those in ≥95 percentile (those who have published three or more SRs in past 10 years) were more likely to publish non‐Cochrane reviews. There was no significant relationship between those experiencing barriers in publishing with Cochrane and the number of Cochrane and/or non‐Cochrane reviews published in 10 years. One would expect to see a high number of Cochrane SRs among those who experienced facilitators. Despite a significant relationship between facilitators and publishing non‐Cochrane SRs, it seems the high‐impact factor and rigorous methodological processes of Cochrane reviewing could be the trade‐off point between publishing a Cochrane vs non‐Cochrane review. Reducing the length of time in publishing SRs and maintaining the rigorous process of conducting SRs has been one of the ongoing discussions among Cochrane authors [14]. In this study, it appears that authors place high value on the visibility and relevance of Cochrane research and thus continue to publish with Cochrane despite the barriers that they encounter. Their responses regarding future publication practices also highlights this.

Even though the downward trend of publishing with Cochrane compared to non‐Cochrane is not associated with the barriers that authors experienced, it is crucial to consider their concerns to strengthen the research capacity‐building initiatives through conducting SRs and the growth of Cochrane in SSA [5, 7]. As South Africa and Nigeria comprised over 70% of the survey authors, which is not surprising as most of the SSA authors reside in these two countries [7], they subsequently have a higher percentage of those who have experienced barriers in publishing with Cochrane. It is vital that these leading nations on SRs in SSA continue to demonstrate capacity building regarding conducting SRs in the region [5]. Addressing the concerns raised may increase their enthusiasm and influence in helping other countries to build capacity in the context of conducting more SRs in SSA. Cochrane recognises some of the barriers that authors faced in publishing SRs, which include the difficult choices that are required in the trade‐off between rigor and speed, and related editorial practices [14]. Cochrane also acknowledges that some SRs use complex questions that require considerable thought and effort, and seeks to continue developing new methodologies to improve the validity and usefulness of reviews that would inform better health decisions. The new editorial structure addresses some of these concerns [14].

The qualitative findings not only inform the questionnaire survey but also provide insights on specific individual experiences that are also essential. The findings show that institutional demand for research output can motivate to publishing non‐Cochrane reviews. Publishing outside Cochrane may encourage diversity of research practices and may also contribute to the mentorship of other authors who may not have been trained using Cochrane methodologies [7]. However, some authors complain that not all reviewers outside Cochrane are methodologically sound when peer‐reviewing synthesis research. In other words, the length of time to complete a SR with Cochrane is not a call to undermine the rigorous research process, rather it is a call to explore ways to reduce the peer reviewing time but also maintain the rigorous reviewing process. This could mean increasing the number of those involved in the reviewing process and providing oversight of the Cochrane Review groups as many authors suggested. Such oversight could start by evaluating the title registration process, which half of the authors agreed to be complicated. Secondly, reflecting on the large proportion of authors who mentioned the lack of adequate funding is an area to also explore to determine the feasibility of directing resources to particular areas. Proper resource allocation in research initiatives is proven to be crucial in health outcomes in SSA [15].

Finally, based on the survey results, most authors disagree that Cochrane does not support the creative ideas of authors in SSA, as they believe that some level of academic freedom should be maintained. The disaccord between authors and journals regarding topics, writing styles, timelines, and deliverables are not unique to Cochrane, but may explain why authors may conduct both Cochrane and non‐Cochrane reviews. Authors residing in specific regions of the world would likely have deeper understanding about the unique health problems in these regions that need to be explored [16] and within specific timelines [17]. However, a mismatch between Cochrane Review group priorities and regional/author priorities seems to exist. Expedited reviews, funding allocation to certain areas, and how the research should be disseminated are key areas to be addressed.

## 6 LIMITATIONS

Although there is some level of triangulation of the findings**—**using both qualitative and quantitative methods, the survey draws on a small number of authors. Despite recommended strategies from research, including re‐checking emails and extending response deadlines, we still had a low response rate. We might have seen differences in the findings, if we had higher participation and response rates. Therefore, in future research, it would be worth exploring a respondent‐driven sampling or snowball sampling as well as drawing on a more comprehensive method of integrating mixed methods data. In the survey, most of the responses recorded were from authors in South Africa and Nigeria. Despite that most of the authors in SSA reside in these countries, there were five of them who responded that they resided outside of SSA at the time of the survey. We could not determine if they expressed these views while residing in or out of SSA.

## 7 CONCLUSION

The findings in this study show that despite barriers that Cochrane authors in SSA encounter in publishing Cochrane Reviews, they continue to publish with Cochrane. This may suggest that authors considered the visibility and relevance of Cochrane research as a trade‐off point. However, the concerns raised by many authors regarding the protracted time of completing a Cochrane Review and the editorial processes require more focused attention. It could be helpful to explore more ways to reduce the peer‐reviewing time while maintaining the rigorous reviewing process.

## AUTHOR CONTRIBUTIONS

**Idriss I. Kallon**: Data curation; formal analysis; investigation; methodology; software; visualization; writing—original draft; writing—review and editing. **Taryn Young**: Methodology; resources; supervision; validation; visualization; writing—review and editing. **Tonya A. MacDonald**: Formal analysis; methodology; validation; writing—review and editing. **Anel Schoonees**: Conceptualization; methodology; writing—review and editing. **Joy Oliver**: Methodology; validation; writing—review and editing. **Dachi I. Arikpo**: Methodology; validation; writing—review and editing. **Solange Durão**: Methodology; validation; writing—review and editing. **Emmanuel Effa**: Methodology; validation; writing—review and editing. **Ameer S.‐J. Hohlfeld**: Methodology; validation; writing—review and editing. **Tamara Kredo**: Methodology; validation; writing—review and editing. **Charles S. Wiysonge**: Methodology; validation; writing—review and editing. **Lawrence Mbuagbaw**: Conceptualization; data curation; methodology; resources; validation; visualization; writing—review and editing.

## CONFLICT OF INTEREST STATEMENT

T. K., S. D., A. H., E. E., C. W., L. M., T. Y., A. S., D. A., and J. O. are all affiliated with Cochrane Centres or on the Steering Group of Cochrane Africa, a registered regional geographic network of Cochrane. T. K. is a member on the Board of Cochrane. C. W. is a member of the Editorial Board of Cochrane Evidence Synthesis and Methods. I. K. and T. M. are not affiliated with Cochrane Centres or on the Steering Group of Cochrane Africa. The views reported here are based on the research findings and do not represent views of Cochrane or its groups.

## PEER REVIEW

The peer review history for this article is available at https://www.webofscience.com/api/gateway/wos/peer-review/10.1002/cesm.12054.

## ETHICS STATEMENT

This study was approved by the Hamilton Integrated Research Ethics Board (project #:8368). All data collection methods were performed in accordance with the relevant guidelines and regulations provided by the aforementioned ethics committee. Only SSA authors who provided informed consent were included in the study. Informed consents were obtained from all study authors. We informed authors that they could leave the in‐depth, semi‐structured interviews or stop the electronic survey at any time. We assured authors that no identifiable data will be shared with anyone except with their consent and the data will be stored in a password‐protected computer in a locked office in the Biostatistics unit of St. Joseph's Healthcare Hamilton, Canada. Only researchers who are part of the project had access to the data.

## Supporting information

Supporting information.

Supporting information.

Supporting information.

## Data Availability

The data that support the findings of this study are available on request from the corresponding author. The data are not publicly available due to privacy or ethical restrictions.

## References

[1] Mulrow CD . Systematic reviews: rationale for systematic reviews. BMJ. 1994;309:597‐599. 10.1136/bmj.309.6954.597 8086953 PMC2541393

[2] Young T , Garner P , Kredo T . Cochrane and capacity building in low and middle‐income countries: where are we at? Cochrane Database Syst Rev. 2013;11:ED000072. 10.1002/14651858.ED000072 PMC10846367 24524153

[3] Mbuagbaw L , Zogo PO , Kredo T , et al. Cochrane Africa: a network of evidence‐informed health‐care decision making across sub‐Saharan Africa. Pan African Med J. 2018;29:196. 10.11604/pamj.2018.29.196.14521 PMC6061814 30061974

[4] Oliver J , Young T . What can the Cochrane collaboration do to support people living in developing countries? A survey. Corroboree Abstracts of the 13th Cochrane Colloquium; 2005. https://abstracts.cochrane.org/2005-melbourne/what-can-cochrane-collaboration-do-support-people-living-developing-countries-survey

[5] Oliver J , Kredo T , Zani B. Barriers and facilitators to completing a cochrane review: a survey of authors in the African region 21st Cochrane Colloquium; 19‐23 September Quebec City, Canada; 2013. https://abstracts.cochrane.org/2013-qu%C3%A9bec-city/barriers-and-facilitators-completing-cochrane-review-survey-authors-african-region.

[6] Schoonees A , Oliver J , Arikpo D , et al. To what extent do Cochrane authors in sub‐Saharan Africa publish Cochrane and non‐Cochrane Reviews? *Youtube*; 2019. https://www.youtube.com/watch?v=fD5Oww3q2Qo&feature=youtu.be

[7] Mbuagbaw L , Schoonees A , Oliver J , et al. Publication practices of sub‐Saharan African Cochrane authors: a bibliometric study. BMJ Open. 2021;11:e051839. 10.1136/bmjopen-2021-051839 PMC8479947 34588260

[8] Coetzee L , Bogler L , De Neve JW , Bärnighausen T , Geldsetzer P , Vollmer S . HIV, antiretroviral therapy and non‐communicable diseases in sub‐Saharan Africa: empirical evidence from 44 countries over the period 2000 to 2016. J Int AIDS Soc. 2019;22(7):e25364. 10.1002/jia2.25364 31353831 PMC6661400

[9] Creswell JW . Research Design: Qualitative, Quantitative, and Mixed Methods Approaches. SAGE Publications; 2009.

[10] O'Cathain A , Murphy E , Nicholl J . Three techniques for integrating data in mixed methods studies. BMJ. 2010;341:c4587. 10.1136/bmj.c4587 20851841

[11] Pluye P , Grad RM , Levine A , Nicolau B . Understanding divergence of quantitative and qualitative data (or results) in mixed methods studies. Int J Mult Res Approaches. 2009;3(1):58‐72. 10.5172/mra.455.3.1.58

[12] McPeake J , Bateson M , O'Neill A . Electronic surveys: how to maximise success. Nurse Res. 2014;21(3):24‐26. 24460562 10.7748/nr2014.01.21.3.24.e1205

[13] Noble H , Mitchell G . What is grounded theory? Evid Based Nurs. 2016;19(2):34‐35. 10.1136/eb-2016-102306 26872777

[14] Chandler J , Cumpston M , Thomas J , Higgins JPT , Deeks JJ , Clarke MJ . Chapter I: Introduction. In: Higgins JPT , Thomas J , Chandler J , Cumpston M , Li T , Page MJ , Welch VA , eds. Cochrane Handbook for Systematic Reviews of Interventions version 6.2 (updated February 2021). Cochrane; 2021. www.training.cochrane.org/handbook

[15] McGillen JB , Anderson SJ , Dybul MR , Hallett TB . Optimum resource allocation to reduce HIV incidence across sub‐Saharan Africa: a mathematical modelling study. Lancet HIV. 2016;3(9):e441‐e448. 10.1016/S2352-3018(16)30051-0 27562745

[16] Berghs M , Ola B , De Chavez AC , Ebenso B . Time to apply a social determinants of health lens to addressing sickle cell disorders in sub‐Saharan Africa. BMJ Glob Health. 2020;5(7):e002601. 10.1136/bmjgh-2020-002601 PMC7388876 32723757

[17] Hsu L , Nnodu OE , Brown BJ , et al. White paper: pathways to progress in newborn screening for sickle cell disease in Sub‐Saharan Africa. J Trop Dis. 2018;06(2):1‐5. 10.4172/2329-891X.1000260 PMC6261323 30505949

