# Supplementary material for: Publication barriers and facilitators of Cochrane authors in sub‐Saharan Africa: A mixed‐methods study
Source: Cochrane Evid Synth Methods. 2024 Apr 3;2(4):e12054. doi: 10.1002/cesm.12054 (PMC11795935; doi:10.1002/cesm.12054)
Supplement: Supplementary file 1 — Supporting information. [file CESM-2-e12054-s002.pdf]

S1: Qualitative interview guide

PUBLICATION PRACTICES OF COCHRANE AUTHORS IN SUB-SAHARAN AFRICA – A MIXED-METHODS STUDY<br><br>QUALITATIVE INTERVIEW GUIDE		
**CATEGORY**	**NO**	**STEPS/QUESTIONS**
**Before the interview**	**1**	Interviewer welcomes interviewee
	**2**	Interviewer introduces himself/herself
	**3**	Ask interviewee if they have any questions about the project
	**4**	Check on interviewee's completion of consent form and ask if they have any questions
**Opening question**	**5**	Please tell me briefly about the work that you do presently.
**Key questions (Factors influencing publication practices)**	**6**	Think back to your research experience, what factors influence your decision to publish a Cochrane Review?
	**7**	What factors influence your decision to publish a non-Cochrane review?
	**8a**	Please explain if you have preference between Cochrane and non-Cochrane Reviews. Probes: If you have experienced barriers in publishing Cochrane Reviews, what do you think are these barriers? Further probes:<br>• Review types<br>• Review teams<br>• Administrative processes (delays)<br>• Mentorship<br>• Review groups
	**9a**	If you have experienced barriers in publishing Cochrane Reviews, what do you think are these barriers? Further probes:<br>• Review teams<br>• Administrative processes (delays)<br>• Mentorship
	**9b**	Do you think your experiences with publishing Cochran reviews would be different if you were from another region of the world? Probes: Please explain how these experiences would be different?<br>• Review teams?<br>• Administrative processes (delays)?<br>• Mentorship?<br>• Others?
	**10**	Have you worked with more than one review group? If yes, please describe your experiences of working with the different review groups. [if no, go to Q11]
	**11**	If you have only worked with one review group, please describe your experiences working with this group. Probes:<br>• Do you think these experiences would be different if you worked with another Cochrane review group?<br>• What would you want to see different with the group you have worked with?
	**12**	Have you every raised a concern about any problems you may have faced publishing Cochrane reviews? Probes:<br>• Where or to whom did you raise this concern?<br>• What kind of response did you receive?<br>• Were you satisfied with such a response?
	**13**	Do you plan to continue publishing Cochrane reviews?
**Concluding question**	**14**	Are there any suggestions you may have to improve on the required processes to complete Cochrane systematic reviews?
	**15**	Is there anything else you would like to speak about regarding your experience with publishing a Cochrane or non-Cochrane review that we did mention?

**Information about interviewee**	**16**	• Number of years as researcher.<br>• Gender<br>• Age<br>• Country of residence
**End of interview**	**17**	Thank you so much for sharing your experiences and ideas with us.
