# Supplementary material for: Publication barriers and facilitators of Cochrane authors in sub‐Saharan Africa: A mixed‐methods study
Source: Cochrane Evid Synth Methods. 2024 Apr 3;2(4):e12054. doi: 10.1002/cesm.12054 (PMC11795935; doi:10.1002/cesm.12054)
Supplement: Supplementary file 2 — Supporting information. [file CESM-2-e12054-s001.pdf]

S2: Quantitative survey questions

PUBLICATION PRACTICES OF COCHRANE AUTHORS IN SUB-SAHARAN AFRICA – A MIXED-METHODS STUDY<br>QUANTITATIVE SURVEY						
	**DEMOGRAPHICS AND RESEARCH EXPERIENCE**					
**No**	**Questions**	**Variables**				
**1**	**Gender**	Male	Female	Other		
**2**	**Country of residence**					
**3**	**Number of years as a researcher**					
**4**	**Number of Cochrane Reviews published in past 10 years (2011-2021)**					
**5**	**Number of non-Cochrane reviews published in past 10 years (2011-2021)**					
**6**	**Year of publication of first Cochrane Review**					
**7**	**Year of publication of first non-Cochrane review**					
	**BARRIERS FOR PUBLISHING COCHRANE REVIEWS**<br>**Describe your level of agreement with the following statement on barriers to publishing Cochrane Reviews**					
	**Questions**	**Variables**				
	**I would rather publish a non-Cochrane Review because**	Strongly disagree	Disagree	Neutral	Agree	Strongly agree
**8**	Cochrane's reviewing process takes too long					
**9**	The Cochrane title registration process is too complicated					
**10**	There are inconsistencies between Cochrane review groups					
**11**	Cochrane's reviewing process limits academic freedom					
**12**	There is lack of support of creative ideas among academics in sub-Saharan Africa					
**13**	There is lack of adequate funding with Cochrane					
**14**	There is lack of transparency in the reviewing process					
**15**	Cochrane does not publish the type of research that we conduct					
**16**	List any other barriers not described above					
	**FACILITATORS FOR PUBLISHING COCHRANE REVIEWS**<br>**Describe your level of agreement with the following statement on factors that would motivate you to publish a Cochrane Review**					
	**Questions**	**Variables**				
**17**	**Have you ever experienced facilitators to publishing Cochrane reviews?**	Yes	No			
	**Questions**	**Variables**				
	**I publish with Cochrane due to:**	Strongly disagree	Disagree	Neutral	Agree	Strongly agree
**18**	Its high-impact factor					
**19**	The rigorous research and peer reviewing process					
**20**	My mentorship duties with students					
**21**	The good mentorship received from Cochrane					
**22**	The good training received from Cochrane					
**23**	The adequate funding received from Cochrane					
**24**	The adequate funding received from other sources					
**25**	Cochrane publishes the types of research that we conduct					
26	List any other facilitators not described above					
	**THE COCHRANE PUBLICATION PROCESS**					
	**Questions**	**Variables**				
**26**	**Do you believe the Cochrane Review publication process can be improved?**	Yes	No			
	**The following are some suggestions on how to improve the Cochrane publication process. Please describe your level of agreement with the following statements**					
	**Questions**	**Variables**				

	**Cochrane needs to:**	Strongly disagree	Disagree	Neutral	Agree	Strongly agree
**27**	Find a balance between rigour and speed regarding the reviewing process					
**28**	Increase the number of reviewing staff					
**29**	Provide more funding to review groups					
**30**	Centralise reviewing processes					
**31**	Increase academic freedom among sub-Saharan Africa authors					
**32**	Provide oversight and functioning of review groups					
**33**	**Please provide additional suggestions here**					
