# Supplementary material for: Publication barriers and facilitators of Cochrane authors in sub‐Saharan Africa: A mixed‐methods study
Source: Cochrane Evid Synth Methods. 2024 Apr 3;2(4):e12054. doi: 10.1002/cesm.12054 (PMC11795935; doi:10.1002/cesm.12054)
Supplement: Supplementary file 3 — Supporting information. [file CESM-2-e12054-s003.PDF]

S3: A Good Reporting of A Mixed Methods Study (GRAMMA) checklist

Guideline	Section: Description and page
Describe the justification for using a mixed methods approach to the research question	We conducted a mixed methods study to provide richer data and to enhance better understanding of the research questions. See heading ***Study setting and design*** **under** ***Methods,*** **page 5.**
Describe the design in terms of the purpose, priority and sequence of methods	We conducted an exploratory sequential study that consisted of two phases: an initial qualitative phase followed and informed by a quantitative phase. This enables the research team to capture relevant topics in the survey for generalizability of the qualitative findings. See heading ***Study setting and design*** **under** ***Methods*****- page 5-7.**
Describe each method in terms of sampling, data collection and analysis	In the qualitative phase, we purposefully selected 12 authors from different sub-Saharan Africa (SSA) geographical zones with different levels of experience in conducting and publishing SRs. We invited all 187 Cochrane authors in SSA who had published at least one Cochrane Review and one non-Cochrane review in the past 10 years as lead or senior author, and who had been identified in the bibliometric study, to participate in a survey. We explained the different data collection techniques and types of analyses conducted. **See sub-heading** ***Sampling and data collection*** **under** ***Methods*** **-page 6-8.**
Describe where integration has occurred, how it has occurred and who has participated in it	We triangulated reasons for preferring non-Cochrane over Cochrane reviews. We explained areas where qualitative and quantitative data converged and where they did not. We explained the number of authors that agreed on key qualitative findings in the survey. See heading ***Triangulating key qualitative and quantitative results*** **– page 15 and page 23**. We also explained more subjective experiences that were useful in qualitative findings that the survey did not capture. **See heading** ***Discussion*** **– page 15-17.**
Describe any limitation of one method associated with the present of the other method	In triangulating the findings we were able to determine that some findings from the qualitative data were not confirmed by participants in the survey. See heading ***Triangulating key qualitative and quantitative results*** **– page 15 and page 23**. On the other hand, we were able to determine strengths of the qualitative data in describing individual experiences that were also important to capture. **See heading** ***Discussion*** **– page 16-17**.
Describe any insights gained from mixing or integrating methods	In triangulating the findings, it provides us with richer data and a better understanding of the barriers and facilitators of Cochrane authors in SSA. See heading ***Triangulating key qualitative and quantitative results*** **– page 15, page 23 and** ***Discussion*** **– page 15-17**

Developed by: O'Cathain, A., Murphy, E., and Nicholl, J. (2008) 'The quality of mixed methods studies in health services research', Journal of Health Services Research and Policy, vol. 13, no. 2, pp. 92-98
